# Association between nut consumption and mortality among Chinese older people: A national cohort study based on CLHLS from 2008 to 2018

**DOI:** 10.3389/fnut.2022.1080714

**Published:** 2022-12-08

**Authors:** Dengxin He, Zheng Huangfu, Minghao Pan

**Affiliations:** ^1^School of Nursing, Hubei University of Chinese Medicine, Wuhan, China; ^2^School of Journalism and Communication, Nanjing Xiaozhuang University, Nanjing, China; ^3^School of Public Health, Wuhan University, Wuhan, China

**Keywords:** nuts, dietary intake, cohort study, mortality, Chinese, COX analysis

## Abstract

**Background:**

Few quantitative studies have explored the associations between nut consumption and better health outcomes among a national cohort of community-dwelling older Chinese people. Given the need for more evidence to support the health benefits of nuts among Chinese people, we investigated whether nut consumption was associated with subsequent 10-year mortality.

**Methods:**

We analyzed data from the Chinese Longitudinal Healthy Longevity Survey. The data on nut consumption at baseline were collected using a questionnaire through face-to-face interviews. The vital status and date of death were ascertained by a close family member or village doctor of the deceased participant during the follow-up survey. Cox analyses were performed to explore the association between nut consumption and mortality. Subgroup analyses by age group (<80 or ≥80 years), sex (male/female), activities of daily living (impaired or normal), and physical exercise (yes or no) were performed to assess whether the association between nut consumption and mortality differed across different populations.

**Results:**

The median survival time was 1,302 days for the 11,915 participants with complete information of survival time and nut consumption. The association between nut consumption and mortality was significant after the adjusting for significant factors in the univariate Cox analyses. The hazard ratios were lower in male participants, those who were <80 years old, and those who did not engage in physical exercise at baseline. The association between nut consumption and mortality was not significant among participants with normal activities of daily living.

**Conclusion:**

The association between nut consumption and mortality was not significant among participants who had normal activities of daily living but was significant among participants who had impaired activities of daily living. Including nuts in the diets cloud help to extend the lifespan in older Chinese people, especially those with impaired activities of daily living.

## Introduction

Nuts are a nutrient dense food and have been an important part of mankind's diets since pre-agricultural times (1). Nuts are a natural plant food rich in fat, and the fatty acid content of nuts is advantageous because they are low in saturated fatty acids and rich in unsaturated fats (2). Nuts also have a rich content of other bioactive macronutrients which cloud be excellent sources of protein and often contain high amounts of L-arginine (3). As L-arginine is the precursor of the endogenous vasodilator, nitric oxide (4), nut consumption might help improve individual vascular function. Nuts also are good sources of dietary fiber which can provide 5–10% of daily fiber needs (5). Further, nuts contain large amounts of essential micronutrients, such as folate, antioxidant vitamins, and phenolic compounds, that cloud contribute to improved health status (2, 6, 7). The macronutrient, micronutrient and non-nutrient components of nuts have all been proved to be beneficial to individual better health outcomes. Further, the beneficial dietary role of nuts may be also based on their prebiotic properties (8).Thus, according to the composition of nuts, nuts may have a health effect on individuals and have become an indispensable component of healthy diets in western countries (9).

Quantitative studies in Western settings have shown that nut consumption is associated with better health outcomes. A recent study by Kim et al. (10) established that nut consumption of ≥5 g/day is associated with a lower risk of metabolic syndrome among U.S. adolescents, based on data from the National Health and Nutrition Examination Survey. Additionally, existing researches has recognized the role of nut consumption or factors associated with this nutritional behavior in reducing the risk of cardiovascular diseases (11, 12). Furthermore, a meta-analysis including a large number of studies in Western settings has shown that nut consumption is associated with reduced mortality (13).

Nuts have been suggested to improve brain function in ancient China and were also an important component of Chinese diet. However, few quantitative studies have explored the associations between nut consumption and better health outcomes among a national cohort of community-dwelling Chinese older people (13, 14). Considering the need for more evidence to support the health benefits of nuts among Chinese, we investigated whether nut consumption at baseline was associated with subsequent 10-year mortality and determined whether associations showed significant differences comparing groups by demographic characteristics, health status, or health behaviors using data from the Chinese Longitudinal Healthy Longevity Survey (CLHLS).

## Methods

### Participants

The CLHLS is a nationwide cohort study. A targeted random sample design was adopted, and half of the counties and cities in 23 of 31 provinces in China were randomly selected through a multistage cluster sampling approach to ensure representativeness. The CLHLS was established in 1998, and recruitment of new participants and subsequent follow-up were conducted in 2000, 2002, 2005, 2008, 2011, 2014, and 2018. The CLHLS conformed to the principles outlined in the Declaration of Helsinki and was approved by the Ethical Review Committee of Peking University (IRB00001052–13074). All participants voluntarily agreed to participate in the study and signed an informed consent form at the time of participation. Information was collected through face-to-face interviews by the CLHLS research staff.

**Figure 1:**
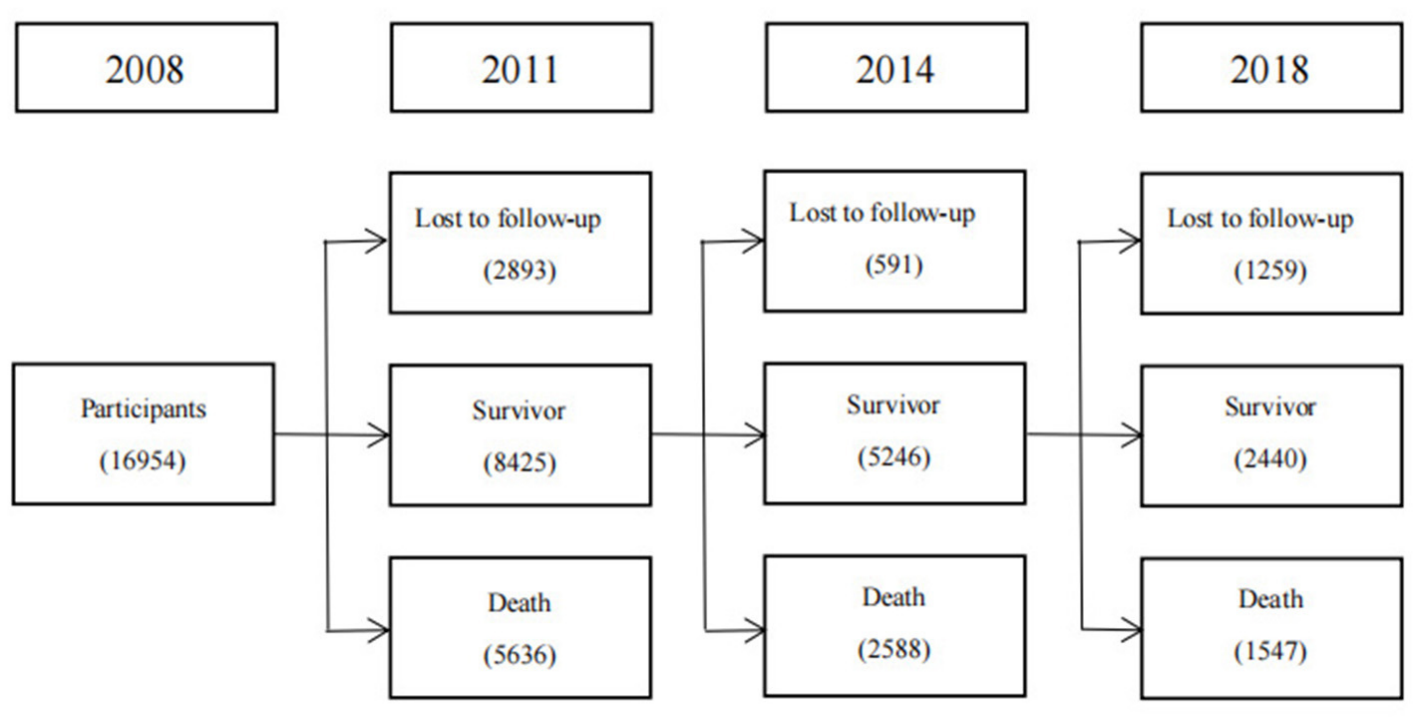
Flowchart.

The current study analyzed data from the 2008 wave of the CLHLS. Follow-up surveys were conducted in 2011, 2014, and 2018. Figure 1 shows how participants in the current study were selected. The 2008 wave included 16,954 older Chinese individuals, and 2,893 participants in 2011, 591 participants in 2011, and 1,259 participants in 2018 were lost to follow-up. Until 2018, 12,211 participants were successfully followed up. For the analysis of the association between nut consumption and mortality, participants who were lost to follow-up or had missing or erroneous information for survival time or nut consumption were excluded, and 11,915 participants were finally included in the survival analysis.

### Measurements

#### Mortality

The outcome was all-cause mortality. The vital status and date of death (for participants who died by the end of the study) were ascertained by a close family member or village doctor of the deceased participant during the follow-up survey conducted in 2011, 2014, and 2018. Participants who survived the last interview were considered censored on the date of their last interviews in 2018. The time interval from the interview date in 2008 until the date of death was considered the duration of follow-up.

#### Nut consumption

The important independent variables was nut consumption which was assessed during the 2008 wave. The trained research staff collected participants' self-reported information on food consumption through face-to-face interviews in the CLHLS (15). Participants were asked to report their frequency of nut consumption, including peanuts, walnuts, chestnuts, or melon seeds. The frequency of nut consumption was recorded as “almost every day”, “at last once per week”, “at least once per month”, “not every month, but occasionally”, or “rarely or never”. The current study coded nut consumption as dichotomous variable by labeling participants answering “almost every day”, “at last once per week”, “at least once per month”, or “not every month, but occasionally” as “1 = yes” and “rarely or never” as “0 = no”.

### Covariates

Covariates, including demographic characteristics, health status, and health behaviors, were assessed during the 2008 wave. Demographic characteristics included age, sex, education, place of residence (city, town or rural), living arrangement (in an institution, alone, or with household members), and economic condition (whether financial support was sufficient to pay for daily expenses).

Health status included participants' activities of daily living (ADL), chronic conditions of hypertension and diabetes. The Katz Index scale was used to measure ADL (16). The Katz Index scale includes six items assessing participants' dressing, bathing, toileting, eating, indoor activities, and continence. The Chinese version of Katz Index scale has been report to be reliable and valid (17). Each item has three response options: “independent,” “needs help,” or “dependent.” Impaired ADL was defined as a participant's response of “dependent” or “needs help” to at least one or more activities associated with one of the six items (18). Chronic conditions, including hypertension and diabetes, were assessed based on self-reported physician's diagnosis.

Health behaviors included physical exercise, smoking, and drinking. Physical exercise (yes vs. no) was measured by asking “do you take exercise regularly in the present?”. Smoking (yes vs. no) was assessed by asking “do you smoke at present?” and drinking (yes vs. no) was assessed by asking “do you drink at present?”.

### Data analysis

Firstly, an independent two-sample *t*-test or analysis of variance for continuous variables and Chi-squared test for categorical variables were conducted to compare characteristics by baseline follow-up status. Then, raw mortality was calculated on several factors (sex, education, residence, living arrangement, financial support, ADL, physical exercise, smoking, drinking, hypertension, and diabetes) and baseline nut consumption status was calculated. For the 11,915 participants with complete information of survival time and nut consumption, survival analysis was conducted. We used Kaplan–Meier method to graph survival curves by nut consumption. Moreover, Cox proportional hazards model was used to assess the association between nut consumption and mortality. To decide whether to control for the above covariates, the “significance-test-of-the-covariance” strategy, in which a variable is adjusted if its coefficient is significant, was conducted by adding the covariates in the model one-by-one (19).

Subgroup analyses, by age groups (<80 or ≥80 years), sex (male/female), ADL (impaired or normal), and physical exercise (yes or no)were performed to assess whether the association between nut consumption and mortality differs across different populations.

SPSS software for Windows version 20.0 (IBM Corp, Armonk, New York) was used for data analysis. A *P*-value of 0.05 or less was considered as significant.

## Results

### Descriptive characteristics

Among the 12,211 participants who were successfully followed up, 9,771 participants died during the three follow-up surveys. We compared the baseline characteristics of surviving and dead participants and found that females had higher raw mortality than males. Participants who had an education of 0 year, had impaired ADL, did not engage in physical exercise, and did not eat nut had a higher raw mortality (Table 1).

**Table 1:** Sample characteristic.

	**Study population**	**Status of follow-up**		
		**Surviving**	**Dead**	***t*** **/*χ^2^***
		**(*N =* 2,440)**	**(*N =* 9,771)**	
Age		75.04 (8.272)	91.14 (9.603)	76.066^***^
**Sex**				23.542^***^
Male	5,170	1,139 (22.03)	4,031 (77.97)	
Female	7,041	1,301 (18.48)	5,740 (81.52)	
**Education**				428.750^***^
0 years	7,966	1,168 (14.66)	6,798 (85.34)	
1–6 years	3,223	923 (28.64)	2,300 (71.36)	
More than 6 years	986	344 (34.89)	642 (65.11)	
**Residence**				4.102
Rural	8,063	1,643 (20.38)	6,420 (79.62)	
City	1,691	308 (18.21)	1,383 (81.79)	
Town	2,457	489 (19.90)	1,968 (80.10)	
**Living arrangement**				16.137^***^
Alone	1,796	372 (20.71)	1424 (79.29)	
With household members	10,213	2,050 (20.07)	8,163 (79.93)	
In an institution	202	18 (8.91)	184 (91.09)	
**Insufficient financial support**				4.091^*^
Yes	9,344	1,905 (20.39)	7,439 (79.61)	
No	2,867	535 (18.66)	2,332 (81.34)	
**ADL**				738.414^***^
Impaired	2,787	53 (1.90)	2,734 (98.10)	
Normal	9,423	2,387 (25.33)	7,036 (74.67)	
**Hypertension**				27.059^***^
Yes	2,245	537 (23.92)	1,708 (76.08)	
No	9,699	1,856 (19.14)	7,843 (80.86)	
Don't know	267	47 (17.60)	220 (82.40)	
**Diabetes**				4.343
Yes	244	60 (24.59)	184 (75.41)	
No	11,735	2,340 (19.94)	9,395 (80.06)	
Don't know	232	40 (17.24)	192 (82.76)	
**Physical exercise**				169.345^***^
Yes	3,022	852 (28.19)	2,170 (71.81)	
No	9,189	1,588 (17.28)	7,601 (82.72)	
**Smoking**				53.561^***^
Yes	2,133	549 (25.74)	1,584 (74.26)	
No	10,078	1,891 (18.76)	8,187 (81.24)	
**Drinking**				40.734^***^
Yes	2,159	539 (24.97)	1,620 (75.03)	
No	10,052	1,901 (18.91)	8,151 (81.09)	
**Nut consumption**				465.164^***^
Yes	4,267	1,306 (30.61)	2,961 (69.39)	
No	7,935	1,130 (14.24)	6,805 (85.76)	

* P < 0.05;

^**^P < 0.01;

*** P < 0.001.

### Kaplan–Meier curves and Cox analysis results

**Figure 2:**
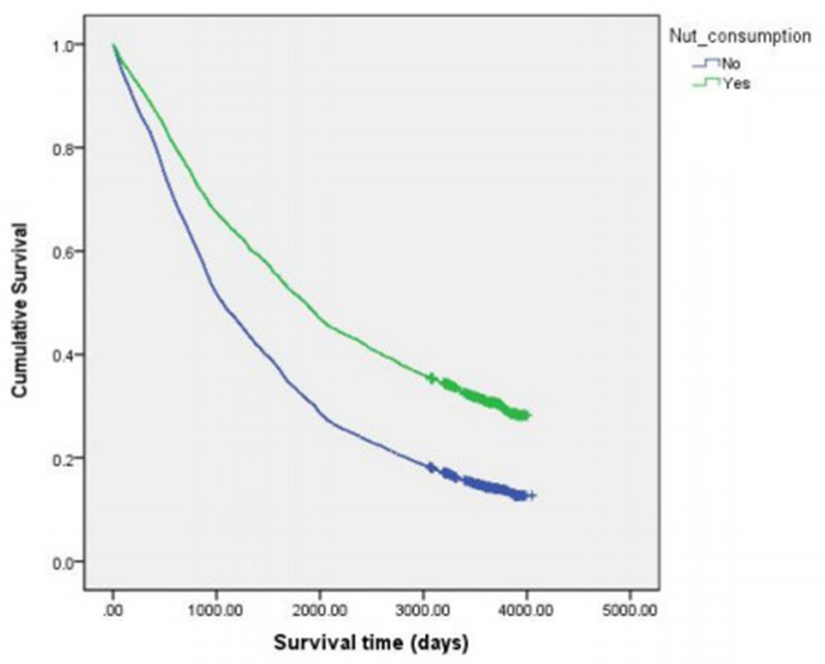
Kaplan-Meier survival curves by nut consumption.

For the 11,915 participants with complete information on survival time and nut consumption, Kaplan–Meier survival curves and Cox analysis were conducted. Kaplan–Meier survival curves represented in Figure 2 illustrate the relationship between nut consumption and a higher probability of survival. The median survival time was 1,302 days for the 11,915 participants with complete information of survival time and nut consumption, 1,060 and 1,863 days for participants who eat nut products and did not eat nut products respectively.

The Cox analysis results are presented in Table 2. The univariate Cox analyses revealed a significant association between nut consumption and mortality (HR = 0.606, 95%CI 0.580–0.633). The results of multivariable Cox analyses showed that the association between nut consumption and mortality was still significant (HR = 0.912, 95%CI 0.872–0.955) after the adjusting for factors that were significant in the univariate Cox analyses (age, sex, education, residence, living arrangement, insufficient financial support, ADL, physical exercise, smoking, drinking, and diabetes).

**Table 2:** The Cox analysis of all variables.

	**Univariate Cox analysis**		**Multivariable Cox analysis**	
	**HR**	**(95% CI)**	**HR**	**(95% CI)**
Age	1.074^***^	1.072–1.077	1.045^***^	1.042–1.047
**Sex**
Male (Ref.)				
Female	1.145^***^	1.009–1.193	1.305^***^	1.245–1.368
Education	0.958^***^	0.952–0.965	0.997	0.993–1.001
**Residence**
Rural (Ref.)
City	1.077^*^	1.015–1.143	1.037	0.974–1.105
Town	0.976	0.928–1.028	1.028	0.976–1.082
**Living arrangement**				
With household members (Ref.)				
Alone	0.926^**^	0.874–0.980	1.027	0.969–1.089
In an institution	1.395^***^	1.201–1.621	1.234^**^	1.061–1.435
**Insufficient financial support**
Yes (Ref.)				
No	0.945^*^	0.903–0.993	1.008	0.252-4.035
**ADL (normal)**
Normal (Ref.)				
Impaired	3.099^***^	2.958-3.247	1.258^***^	1.195–1.325
**Hypertension**
Yes (Ref.)				
No	0.373	0.093–1.488		
**Diabetes**
Yes (Ref.)				
No	0.090^*^	0.013–0.645	0.224	0.031–1.604
**Physical exercise**
No (Ref.)				
Yes	0.671^***^	0.671–0.705	0.892^***^	0.848–0.939
**Smoking**
Yes (Ref.)				
No	0.778^***^	0.737–0.821	0.976	0.919–1.037
**Drinking**
Yes (Ref.)				
No	0.815^***^	0.772–0.860	0.959	0.906–1.017
**Nut consumption**
No (Ref.)				
Yes	0.606^***^	0.580–0.633	0.912^***^	0.872–0.955

* P < 0.05;

** P < 0.01;

*** P < 0.001.

### Subgroup analyses

Table 3 shows the HRs by nut consumption for different subgroups. To assess whether mortality risks by nut consumption differed by age (<80 years old or ≥80 years old), sex (male or female), ADL (impaired or normal), and physical exercise (yes or no), we performed separate multivariable Cox models for each subgroup with full adjustment. The HRs were lower in participants who were <80 years old (HR = 0.744, 95%CI 0.662–0.835), male participants (HR = 0.858, 95%CI 0.802–0.919), and those who did not engage in physical exercise at baseline (HR = 0.865, 95%CI 0.821–0.912) than in participants who were ≥80 years old (HR = 0.866, 95%CI 0.825–0.909), female participants (HR = 0.900, 95%CI 0.847–0.957), and those who engaged in physical exercise at baseline (HR = 0.882, 95%CI 0.804–0.967), respectively. The association between nut consumption and mortality was not significant among participants with normal ADL (HR = 0.923, 95%CI 0.841–1.013), but significant among participants with impaired ADL (HR = 0.884, 95%CI 0.839–0.931).

**Table 3:** Subgroup analyses of association between nut consumption and mortality.

**Subgroup**	**HR**	**95%CI**
**Age**		
< 80 years old	0.744^***^	0.662–0.835
≥80 years old	0.866^***^	0.825–0.909
**Sex**		
Male	0.858^***^	0.802–0.919
Female	0.900^**^	0.847–0.957
**ADL**		
Impaired	0.884^***^	0.839–0.931
Normal	0.923	0.841–1.013
**Physical exercise**		
Yes	0.882^**^	0.804–0.967
No	0.865^***^	0.821–0.912

** P < 0.01;

*** P < 0.001.

## Discussion

In this nationwide cohort study of community-dwelling Chinese older people, nut consumption was associated with an 8.8% lower risk of mortality compared with non-consumption of nuts at baseline. This result is consistent with the finding in most previous researches on this topic in Western countries (14, 20, 21). Wang et al. (20) analyzed data from 6,072 individuals who participated in the National Health and Nutrition Examination Survey and reported that higher nut consumption was significantly associated with lower all-cause mortality in the population without chronic kidney disease and nut consumption of 1–6 times per week was significantly related to lower all-cause mortality in the population with chronic kidney disease. Fernandez-Montero et al. (21) analyzed data from a Spanish cohort and found that participants who consumed nuts twice or more per week had a 56% lower risk for all-cause mortality than those who never or almost never consumed nuts. Despite the differences in the classification of nut consumption and the participants in these studies, nut consumption showed a significant effect on individuals' reduced all-cause mortality. Further, although some researchers reported inconsistent findings that the association between nut consumption and mortality was not quite statistically significant in their study, most of them found lower mortality in participants with high nut consumption compared with participants with low or no nut consumption (22, 23). Our findings add evidence to support the association between nut consumption and decreased mortality among older Chinese people and suggest that these older Chinese people could also benefit from nut consumption.

To the best of our knowledge, the current study is the first to show the differences in the association between nut consumption and mortality in the Chinese elderly population. We found the association between nut consumption and mortality in the subgroup analysis was consistently significant comparing groups by age, sex, and physical exercise. These results suggest that the association between nut consumption and mortality is relatively robust in different groups divided by age, sex, and physical exercise. Interestingly, we found that the association between nut consumption and mortality was not significant among participants with normal ADL. However, we should interpret this result with caution because the finding regarding the association between nut consumption and mortality among participants with normal ADL were trending in the expected direction. And it is suggested that further research to explore the difference in the association between nut consumption and mortality in participants with different ADL needed to be conducted.

The association of nut consumption with decreased mortality could be explained by the health effects of nut consumption on the physical and mental status (24, 25). Firstly, nut consumption has been reported to be associated with improved inflammatory status, including C-reactive protein, interleukin-6, and fibrinogen, and thus reduce risk of cardiovascular disease and type 2 diabetes (26). Secondly, nuts, especially nuts rich in monounsaturated fatty acids, may have health effect on individuals oxidative status (2), and thus reduce risk of many health problems, such as cardiovascular and inflammatory diseases, and cancer (27). Thirdly, diet enriched with nuts may improve insulin sensitivity and fasting glucose levels (28, 29), therefore, nut consumption could contribute to better metabolic status (30, 31), decreased body weight as well as lower body weight gain over time and thus reduce the risk of obesity (32). Fourthly, diet with nuts, such as pistachios, could have favorable effects on vascular reactivity and reduce risk of cardiovascular disease (33–35). Finally, dietary patterns also play an important role in mental health. Higher nut consumption cloud be related to better mood state, fewer depressive symptoms, and a lower risk for depression (25). These mechanisms may contribute to the prevention of cardiovascular, other chronic, and mental diseases, leading to a reduction of all-cause mortality. Both the biological plausibility of nutrients in nuts and the findings of previous researches support the present findings of the health effects of nut consumption on mortality in an older Chinese population with impaired ADL. Thus, though the reason nut consumption was significantly associated with decreased mortality among participants with impaired ADL but not among participants with normal ADL, needs further exploration, including nuts in the diet cloud help extend lifespan in older Chinese, especially those with impaired ADL.

The current study has several strengths. It is among the few studies to explore the association between nut consumption and decreased mortality in older Chinese population. Moreover, the data we used was from the CLHLS, which is a large nationally representative survey. Thus, the results of this study have strong generalizability. Additionally, the findings of this study could add evidence to support the differences in the association between nut consumption and decreased risk of mortality among elder Chinese with different group of age, sex, physical exercise, and ADL. Additionally, the CLHLS contains detailed covariates, including age, sex, education, residence, living arrangement, financial support, ADL, smoking, drinking, and chronic diseases, which allow us to control for a large number of covariates in the multiple Cox model.

This study has several limitations. Firstly, the CLHLS did not include information on causes of death, limiting our ability to perform cause specific analysis. Secondly, we have limited information on nut consumption variables, such as the amount of nuts consumed and the specific type of nuts, limiting our ability to perform more detailed analysis.

## Conclusion

In conclusion, our study explored the association between nut consumption and decreased mortality in a national of community-dwelling older Chinese individuals and specific subpopulations. The association between nut consumption and mortality was significant in overall population and specific subpopulations divided according to age, sex, physical exercise. Furthermore, the association between nut consumption and mortality was not significant among participants who had normal ADL at baseline but significant among participants who had impaired ADL at baseline. Including nuts in the diets cloud help to extend the lifespan in older Chinese people, especially those with impaired ADL.

## Data availability statement

The datasets presented in this study can be found in online repositories. The names of the repository/repositories and accession number(s) can be found below: https://opendata.pku.edu.cn/dataverse/CHADS.

## Ethics statement

The studies involving human participants were reviewed and approved by Ethical Review Committee of Peking University. The patients/participants provided their written informed consent to participate in this study.

## Author contributions

DH undertook the analyses and wrote the first drafts manuscript. MP and ZH critically reviewed the manuscript. All authors read and approved the final manuscript.
